# Mental and Sexual Health Challenges Among Sexual Minority Individuals

**DOI:** 10.1192/j.eurpsy.2025.2321

**Published:** 2025-08-26

**Authors:** A. Stern, A. Gewirtz-Meydan, S. Maoz

**Affiliations:** 1 Occupational Therapy, Ben-Gurion University of the Negev, Beer Sheva; 2 Social Work, University of Haifa, Haifa, Israel

## Abstract

**Introduction:**

Sexual minority individuals face unique challenges in mental and sexual health, emotion regulation, and well-being. Although understanding the complex dynamics among these variables in the context of diverse sexual orientations and gender identities is crucial to supporting and tailoring comprehensive interventions, limited research has investigated their overlapping relationships and intersections.

**Objectives:**

The current study aims to examine the connections between mental health, sexual health, emotion regulation, and well-being among sexual minority and heterosexual individuals in Israel. The main purpose was to provide a comprehensive understanding of the unique challenges sexual minority individuals face.

**Methods:**

The study included 465 participants, 324 (70%) were identified as heterosexual individuals and 119 (26%) as sexual minority individuals. Various variables were assessed using an online anonymous questionnaire, including mental health (anxiety, depression, suicide ideation, substance use disorder), sexual health (sex-related distress, problematic pornography use, compulsive sexual behavior disorder), emotion-regulation, and well-being. Between-group differences were analyzed using Mann-Whitney U tests. Network analysis was conducted to examine the centrality and edges of relationships between variables within each group.

**Results:**

Significant differences were found between the heterosexual and sexual minority groups across the measured variables. Sexual minority individuals reported higher levels of psychopathology, lower sexual health, as well as lower levels of emotion regulation and well-being compared to heterosexual individuals. Network analysis revealed that the number of diagnosed psychopathologies and depression were central nodes in the sexual minority group, while sexual functioning played a central role in the heterosexual group. The sexual minority group’s network showed less stability, suggesting distinct subpopulations within this group.

**Conclusions:**

This study contributes to understanding the unique mental and sexual challenges sexual minority individuals face and the intersections between mental health, sexual health, emotion regulation, and well-being. These findings highlight the importance for mental health professionals to acknowledge and address these connections, emphasizing the need for tailored psychosocial interventions that integrate sexual health.

**Disclosure of Interest:**

None Declared

